# Fetal sex determination in twin pregnancies using non-invasive prenatal testing

**DOI:** 10.1038/s41525-019-0089-4

**Published:** 2019-07-04

**Authors:** Darine Villela, Huiwen Che, Marijke Van Ghelue, Luc Dehaspe, Nathalie Brison, Kris Van Den Bogaert, Koen Devriendt, Liesbeth Lewi, Baran Bayindir, Joris Robert Vermeesch

**Affiliations:** 1 Department of Human Genetics, KU Leuven, Leuven, Belgium; 2 Department of Genetics and Evolutionary Biology, Institute of Biosciences, University of São Paulo, São Paulo, Brazil; 3 Department of Medical Genetics, Division of Child and Adolescent Health, University Hospital of North Norway, Tromsø, Norway; 4 Department of Clinical Medicine, University of Tromsø, Tromsø, Norway; 5 Clinical Department of Obstetrics and Gynecology, University Hospitals Leuven, Leuven, Belgium; 6 Department of Development and Regeneration, Cluster Woman and Child, Group Biomedical Sciences, KU Leuven, Leuven, Belgium

**Keywords:** Genetics research, Medical genetics

## Abstract

Non-invasive prenatal testing (NIPT) is accurate for fetal sex determination in singleton pregnancies, but its accuracy is not well established in twin pregnancies. Here, we present an accurate sex prediction model to discriminate fetal sex in both dichorionic diamniotic (DCDA) and monochorionic diamniotic/monochorionic monoamniotic (MCDA/MCMA) twin pregnancies. A retrospective analysis was performed using a total of 198 twin pregnancies with documented sex. The prediction was based on a multinomial logistic regression using the normalized frequency of X and Y chromosomes, and fetal fraction estimation. A second-step regression analysis was applied when one or both twins were predicted to be male. The model determines fetal sex with 100% sensitivity and specificity when both twins are female, and with 98% sensitivity and 95% specificity when a male is present. Since sex determination can be clinically important, implementing fetal sex determination in twins will improve overall twin pregnancies management.

## Introduction

Non-invasive prenatal testing (NIPT) using cell-free DNA (cfDNA) is changing the standard care in obstetrics. Although the main purpose of NIPT is the screening for the viable autosomal aneuploidies (trisomies 21, 18, and 13), the test was first established in clinical setting for fetal sex determination, based on the presence or absence of Y chromosome sequence in the maternal plasma.^1^ Discrimination of fetal sex is clinically relevant not only for families at high risk of sex-linked disorders, such as hemophilia and Duchenne muscular dystrophy,^2,3^ but also in cases for which the development of external genitalia is ambiguous,^4^ and in some endocrine disorders like congenital adrenal hyperplasia, where there is a masculinization of the female fetus that can be preventable with antenatal treatment.^5^ It is noteworthy mentioning that the assessment of fetal sex through NIPT offers earlier test results compared to ultrasound, and has been shown to reduce the number of invasive procedures significantly.^6,7^

Currently, prediction of fetal sex in NIPT is performed using either real-time PCR or Y chromosome read counting.^8,9^ In both methods, the fetus is presumed to be male or female based on whether Y chromosome material is detected or not. It has been demonstrated that the performance of NIPT for aneuploidy detection is best achieved in singleton pregnancies when compared to multiple gestation.^10,11^ However, little information is available about the precision of NIPT for fetal sex determination in twins. Since standard sequential screening tests are not very precise in multiple gestation, and considering the increased risks for complications associated with this type of pregnancy, it is important to develop accurate non-invasive methods to identify aneuploidy and sex discordances in twins to improve overall pregnancy management. Moreover, the rate of multiple gestation has dramatically increased over the last few years. While the incidence of monozygotic twins is about 3–5 per 1000 births, the rate of dizygotic twins varies with maternal age, gravidity, ethnicity, familial history, and the use of assisted reproductive techniques.^12,13^ In Europe and the US for example, the incidence of dizygotic twins corresponded to 8 per 1000 births, but mainly due to in vitro fertilization (IVF) the incidence is rising to 16–30 per 1000 births today.^12,14^ Also, it has been estimated that approximately 24% of successful IVF procedures result in multiple gestation, increasing even the risk of monozygotic twins.^14^

Despite NIPT being considered a feasible option for risk estimation of fetal aneuploidies in twin pregnancies, it is well-known that its accuracy depends on the proportion of fetal cfDNA circulating in the maternal plasma.^15^ Even though the circulating cfDNA has shown to be higher in twin pregnancies compared with singletons, the fetal fraction per twin can be lower.^10,11^ In addition, the relative proportion of fetal DNA per twin might be different, which could lead to an erroneous result in both sex or aneuploidy determination because one of the twins might contribute to an unsatisfactory fetal fraction. Notably, chorionicity may also impact NIPT results since cfDNA is placental in origin, resulting from apoptotic trophoblasts,^16^ and the representation of fetal fraction could be affected depending on whether the twins share the same placenta or not. Previous studies have indeed shown that prenatal aneuploidy screening is less robust in twin pregnancies, and regardless the high detection rate for fetal sex determination, discordant results still occur.^17,18^ Especially in dizygotic twins, depending on the amount of Y chromosome present in the maternal circulation it might be challenging to precisely predict mixed-sex pairs. Recently, we introduced a novel approach to map mosaicism which can accurately predict the presence of discordant autosomal constitution in twin pregnancies.^19^ Expanding on this approach, we present a new data analysis tool that allows the accurate prediction of fetal sex in twin pregnancies.

## Results

**Fig. 1:**
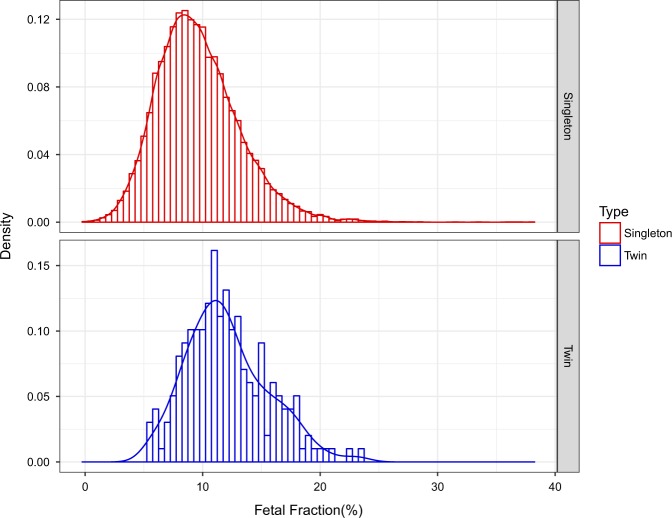
Distribution of fetal fraction. The histograms show the overall distribution of fetal fraction estimation in 21,912 singleton pregnancies (upper panel) and 198 twin pregnancies (lower panel). The dataset of fetal fraction used to plot the normal distribution in singleton pregnancies was the same presented in Brison et al.^19^

We collected cfDNA sequencing data from 198 twin pregnancies. We applied a multinomial logistic regression using the normalized frequency of X and Y chromosomes combined with fetal fraction estimation to predict fetal sex in twins. Table 1 presents the statistical parameters of NIPT sequencing data regarding the total read counts as well as the normalized frequency of sex chromosomes for all the samples. A comparison of an overall fetal fraction distribution in singleton and twin pregnancies is presented in Fig. 1. In the twins dataset, fetal fraction ranges from 5.4% to 23.5%. The distribution of fetal fraction in twins is centered at higher values with an average of 12.1% compared to 9.6% in singleton pregnancies (singleton pregnancies: 9.64 ± 3.52; twin pregnancies: 12.08 ± 3.50; Wilcoxon rank-sum test *p* < 2.2 × 10^−16^).

**Table 1:** Statistical parameters of NIPT sequencing data

	Read count			Normalized frequency		
	Mean	SD	Median	Mean	SD	Median
FF						
chr X	2692.978	26.336	2687.227	4.973E−02	4.806E−04	4.964E−02
chr Y	2.650	0.198	2.615	4.894E−05	3.668E−06	4.833E−05
FM						
chr X	2595.761	42.038	2590.694	4.793E−02	7.379E−04	4.786E−02
chr Y	7.733	1.581	7.320	1.428E−04	2.926E−05	1.350E−04
MM						
chr X	2495.528	83.748	2503.867	4.607E−02	1.518E−03	4.617E−02
chr Y	13.165	3.858	12.579	2.431E−04	7.137E−05	2.324E−04

Because of homologous regions between X and Y chromosomes, a number of reads in female pregnancies systematically maps to the Y chromosome. Average of total number of reads generated in shallow whole-genome sequencing = 16,198,687. Normalized X and Y chromosome read counts were corrected by GC content, which largely reduced the effect of inequality of total number of reads generated across samples

*FF* female–female, *FM* female–male, *MM* male–male

**Fig. 2:**
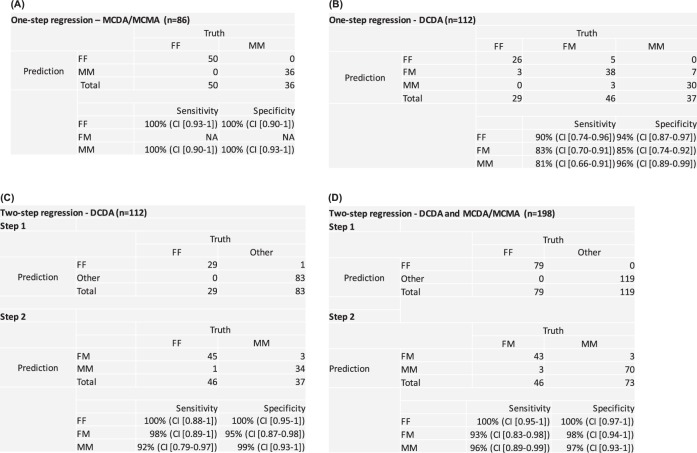
Multinomial logistic regression model to predict fetal sex in twin pregnancies. A one-step regression was applied in MCDA/MCMA **a** and DCDA samples **b** separately. A second-step regression was applied in DCDA samples if a twin pregnancy was classified as non-FF to predict whether the twin sex is FM or MM **c**. The same strategy was used analyzing DCDA and MCDA/MCMA samples together **d**. The sensitivity and specificity were calculated based on the prediction results. DCDA dichorionic diamniotic, MCDA monochorionic diamniotic, MCMA monochorionic monoamniotic, FF female–female, FM female–male, MM male–male, 95% confidence interval (CI)

A one-step regression was first applied to determine fetal sex as being female–female (FF), female–male (FM), or male–male (MM). In DCDA twin pregnancies, the pairs can be either monozygotic or dizygotic. For the latter, there are three possible sex combinations, i.e., FF, FM, or MM. In contrast, MCDA/MCMA is a subset of monozygotic twin pregnancies, and evidently can only be either FF or MM. This analysis was first performed in DCDA and MCDA/MCMA samples separately (Fig. 2). In MCDA/MCMA twins, the algorithm applied presents 100% sensitivity and 100% specificity for FF and MM discrimination (95% CI [0.93–1] and 95% CI [0.90–1]) (Fig. 2a). However, in DCDA twins, sensitivity and specificity are diminished due to a possibility of a mixed-sex pair (FM) (90% sensitivity [95% CI [0.74–0.96]] and 94% specificity [95% CI [0.87–0.97]] for FF; 83% sensitivity [95% CI [0.70–0.91]] and 85% specificity [95% CI [0.74–0.92]] for FM; 81% sensitivity [95% CI [0.66–0.91]] and 96% specificity [95% CI [0.89–0.99]] for MM) (Fig. 2b).

**Fig. 3:**
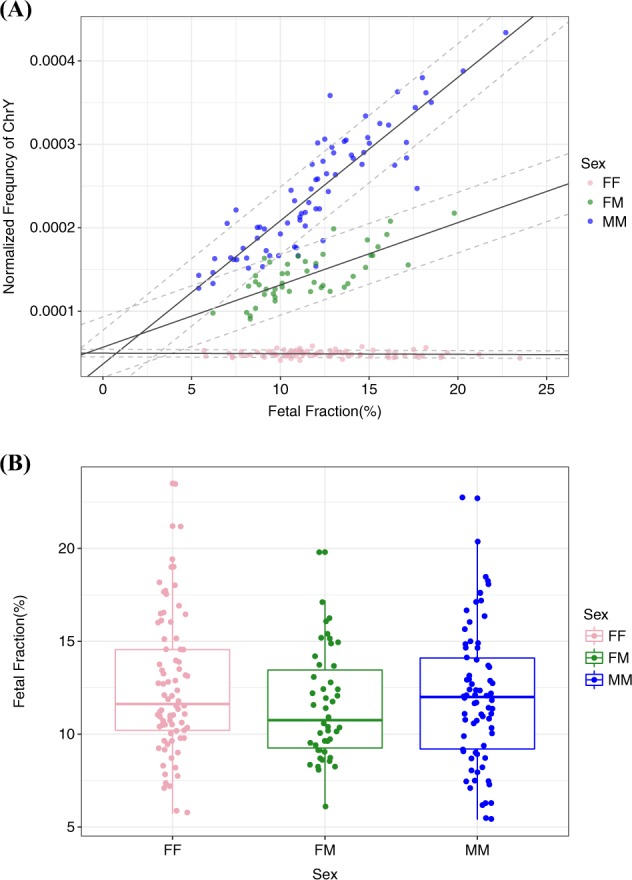
Fetal fraction correlation in twin pregnancies. **a** Correlation of fetal fraction estimation with the normalized frequencies of Y chromosome reads in twin pregnancies. Full lines and dashed lines represent the average and ±3*standard deviation (s.d.) of fetal fraction estimation, respectively. **b** Comparison of fetal fraction among the three different sex groups in DCDA twin pregnancies. FF female–female, FM female–male, MM male–male

To improve the accuracy of our test we applied a second-step regression in the DCDA samples. Because discrimination of fetal sex relies mostly on the presence or absence of Y chromosome, it is possible to predict with high accuracy whether one of the pairs are male or not in the dizygotic twins. Hence, when the Y chromosome is present the twins can be assumed to be non-FF. Based on this assumption, if the sample is classified as non-FF, a second-step regression was used to predict whether the twin sex is FM or MM. Using this strategy, we were able to discriminate fetal sex with 98% sensitivity (95% CI [0.89–1]) and 95% specificity (95% CI [0.87–0.98]) in the FM group, and with 92% sensitivity (95% CI [0.79–0.97]) and 99% specificity (95% CI [0.93–1]) in the MM group. (Fig. 2c). Four cases of discordant results occurred; one FM case was predicted to be MM, and three MM cases were predicted to be FM. Of note, by incorporating fetal fraction estimation into the regression model a better accuracy was achieved for fetal sex determination (Supplementary Data 1). Plotting the normalized frequencies of Y chromosome reads in function of fetal fraction in the three groups, we can extrapolate the minimum fetal fraction required to discriminate twin sex. Based on the intersecting lines of the three groups, the threshold for discrimination of FM and MM is a fetal fraction of 2.08%, and for MM and FF a fetal fraction of 0.76% (Fig. 3a). This is the theoretical minimum. However, when taking the variation in the normalized Y chromosome reads into account, one can assume that sex determination is accurate for all pregnancies when the fetal fraction is above 10% and will be accurate in over 50% of the cases when above 6%. We also wondered whether the fetal fraction estimation would be different in twin pregnancies with only female fetuses, only male fetuses of male–female fetuses. As expected, no significant difference was observed between these groups (Fig. 3b).

**Fig. 4:**
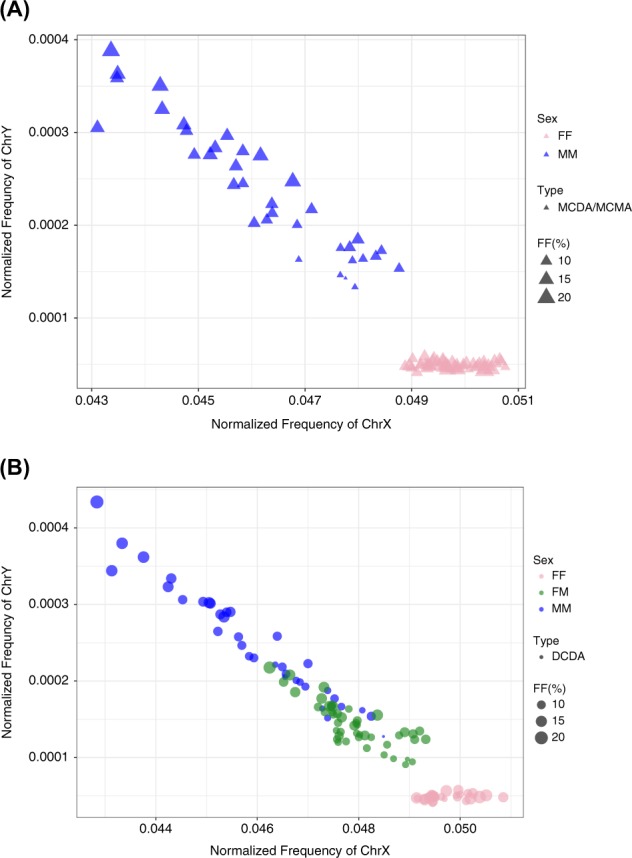
Performance of multinomial logistic regression model to predict fetal sex in twin pregnancies. Fetal fraction estimation of each sample is presentedas % based on SeqFF method. Each icon size correspond to a representation of fetal fraction estimation (10%, 15%, and 20%) in which the DNA samplesare scaled accordingly. One-step regression analysis was used to discriminate fetal sex in MCDA/MCMA twin pregnancies **a**. A two-step regressionanalysis was applied in DCDA samples separately **b**. DCDA dichorionic diamniotic; MCDA monochorionic diamniotic; MCMA monochorionic monoamniotic; FF female–female; FM female–male; MM male–male

**Fig. 5:**
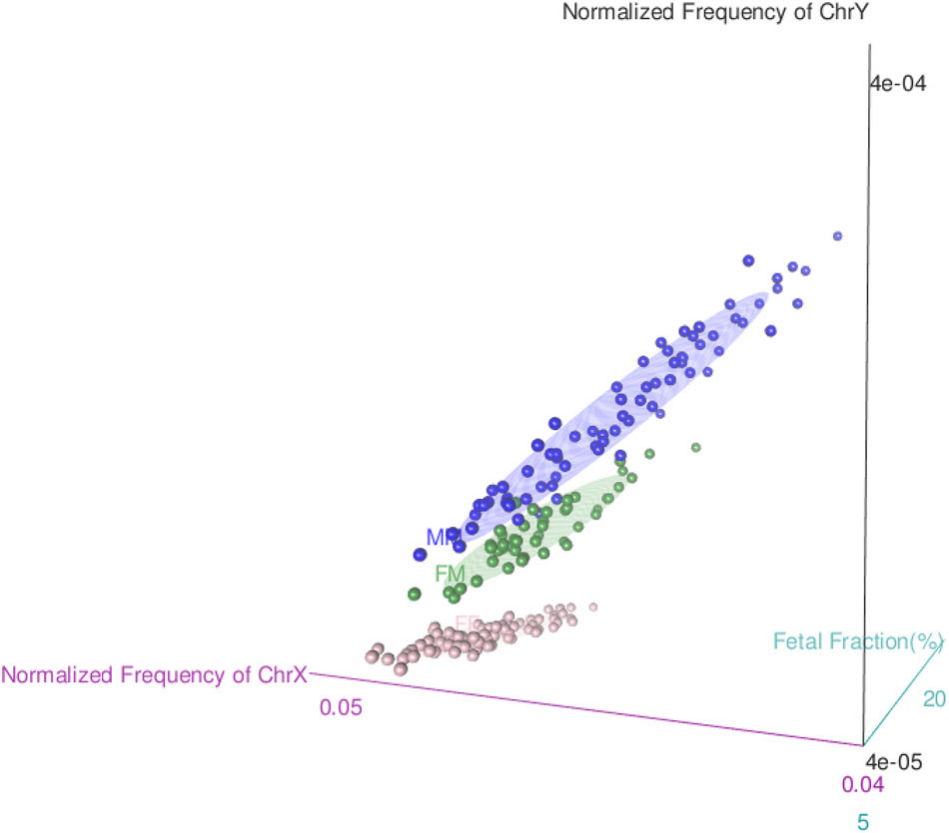
3D plot of the multinomial logistic regression model’s performance to predict fetal sex in twin pregnancies. Correlation of the three parameters incorporated in the regression model (i.e., normalized frequencies of chromosome X and Y reads, and fetal fraction estimation) among the different sex categories in all 198 twin pregnancies without the discrimination of chorionicity. FF female–female, FM female–male, MM male–male

Figure 4 shows the performance of our analysis in all twin pregnancies. All plots present a negative correlation between the normalized frequency of X chromosome against the Y chromosome, and the FF group created clusters clearly different from the others. This evidence points out to the efficiency of using a second-step regression analysis. Also, fetal fraction estimation of each sample is presented in the plots. The icon sizes correspond to a representation of the fetal fraction. We also tested whether the accuracy of our model would be impacted in cases where it is not possible to obtain chorionicity information of the twin pregnancies before NIPT analysis. Analyzing all 198 samples together, the sensitivity and specificity were slightly lower compared to the DCDA samples evaluated separately; 93% sensitivity (95% CI [0.83–0.98]) and 98% specificity (95% CI [0.94–1]) in the FM group, and 96% sensitivity (95% CI [0.89–0.99]) and 97% specificity (95% CI [0.93–1]) in the MM group (Fig. 2d). The 3D plot helps visualize the correlation of the three parameters incorporated in the regression model among the different sex categories (Fig. 5) (3D rotation animation is presented in Supplementary Movie 1). Hence, when chorionicity is not known, the sex of six twins were misclassified: three FM cases were predicted to be MM, and three MM cases were predicted to be FM.

## Discussion

Despite the high accuracy of NIPT in singleton pregnancies, there are relatively few studies about its efficiency in multiple gestations. Moreover, the majority of those studies focus on the screening of the common aneuploidies^10,11^ and little data is available about the precision of NIPT for fetal sex discrimination in twins. Here, we present an accurate sex prediction model to discriminate fetal sex both in DCDA and MCDA/MCMA twin pregnancies. All of the 86 MCDA/MCMA twin sexes were correctly classified, and 108 out of the 112 DCDA twin sexes were correctly classified. Hence, the overall sex classification accuracy is 98%, with an overall sensitivity and specificity of 100% when only female fetuses are present, and with 98% sensitivity and 95% specificity when a male is present.

In monochorionic twins, the two monozygotic (genetically identical) fetuses contribute the same alleles of cfDNA into the maternal circulation, and NIPT analysis can be carried out with the same accuracy as in singleton pregnancies. In dichorionic twins, the majority of pregnancies are dizygotic. Recently, Milan and colleagues investigated the accuracy of a sex prediction model based on the normalized chromosome value (NCV) of the sex chromosomes by linear regression.^20^ Without discriminating between mono- or dichorionic pregnancies, the authors reported a detection rate of 98% for fetuses sex classification with a sensitivity and specificity of 97% and 95%, respectively. Here, we show that a two-step linear regression with incorporation of fetal fraction in the model increases the performance for sex determination. We also demonstrate that knowledge of the chorionicity helps in the classification. Incorporating this information in the analysis, gives the optimal results. However, it is not always possible to obtain information about chorionicity of the twin pregnancies before NIPT analysis. Therefore, we also determined how the accuracy would be impacted when excluding this information from the analysis. When we analyzed all 198 samples together, the sensitivity and specificity were slightly lower compared with the analysis of DCDA pregnancies only.

Four cases of discordant results occurred; one FM case was predicted to be MM, and three MM cases were predicted to be FM. A first potential reason for a wrong sex determination could be a low fetal fraction. In our dataset, the lowest fetal fraction is 5.4% in male twins and this sample is correctly classified. Thus, a low fetal fraction is not the reason for the false positive results. Nevertheless, we observe that for male twins with lower fetal fraction and mixed sex cases with higher fetal fraction, the sex determination is decreased. Hence, the fetal sex determination is not necessarily higher with increased fetal fraction. A second potential reason for discrepant results could be unequal shedding of DNA from the two placentas in the maternal blood circulation. Studies analyzing maternal plasma cfDNA in dizygotic twins have shown that the circulating fetal DNA from both placentas can vary nearly 2-fold.^21^ Accordingly, it is possible that in a dizygotic twin pregnancy the fetal fraction of one fetus may be too low for reliable cfDNA analysis. This could lead to an erroneous result for fetal sex as well as for aneuploidy determination. We thus reiterate that the performance of the test is not necessarily only linked to the overall/combined fetal fraction since different fetal cfDNA contributions in the DCDA group could impact performance in those cases. Another likely cause for discrepancies is the presence of X chromosome copy number mosaicism in the maternal blood. Several studies show that imbalances in the maternal genome impair NIPT precision.^22,23^ Sex chromosomal mosaicism can also interfere with fetal sex chromosome determination in singleton pregnancies.^24,25^ Finally, we cannot exclude the presence of fetal sex chromosome aneuploidies (SCAs). Interestingly, our approach in analyzing the sex in twin pregnancies shows similarities with the method described to discriminate SCAs in singleton pregnancies. Mazloom and colleagues presented an algorithm based on normalized sequence reads of sex chromosomes as well, that detected SCAs in singleton pregnancies with a combined sensitivity of 96.2% and a false positive rate of 0.3%.^26^ Unfortunately, we consider that the interpretation of SCAs in multiple pregnancies is not possible with the currently available methods.

In summary, the accurate sex prediction model demonstrated here, based on the normalized frequencies of X and Y chromosomes and fetal fraction estimation, represents a valuable approach to characterize fetal sex in twins, and can help improve overall twin pregnancies management. Nevertheless, despite the high precision of the prediction a limited level of uncertainty remains. Hence, confirmation by ultrasound is warranted.

## Methods

### Sample collection and processing

This retrospective study was granted by the Ethical Committees of the University Hospital Leuven, and a written informed consent was obtained from all participants. NIPT sequence reads were recovered from twin pregnancies with documented sex. Fetal sex confirmation was done either by ultrasound during the 20th week of gestation or postnatally by visual inspection. Table 2 presents the characteristics of all samples included in this research. In total, 198 twin pregnancies were investigated. Chorionicity and amnionicity were determined by ultrasound in all twin pregnancies, which are classified as dichorionic diamniotic (DCDA), monochorionic diamniotic (MCDA), or monochorionic monoamniotic (MCMA), based on whether the pairs share the same placenta and amniotic sac or not. Genetic counseling is provided by the gynecologists before sampling for NIPT. The patient is informed that in case of a dizygotic twin pregnancy, the accuracy of the test for fetal sex determination and aneuploidy detection can be lower compared to a monozygotic twin pregnancy due to a possibility of a mixed-sex twin. Peripheral blood samples from pregnant women were collected from 10 weeks of gestation onwards in either cell-free DNA BCT tubes (Streck, Omaha, NE, USA) or cell-free DNA collection tubes (Roche Diagnostics, Germany). Cell-free DNA extraction, DNA library preparation, as well as whole genome sequencing were carried out as previously described.^27,28^

**Table 2:** Twin characteristics

	DCDA (*n* = 112)	MCDA/MCMA (*n* = 86)
Clinical phenotype (twin A/B)		
46,XY/46,XY	37	36
46,XY/46,XX	46	
46,XX/46,XX	29	50

*DCDA* dichorionic diamniotic, *MCDA* monochorionic diamniotic, *MCMA* monochorionic monoamniotic, *NIPT* non-invasive prenatal testing

### Fetal sex determination in twin pregnancies using NIPT

NIPT analysis was performed as described in Bayindir et al.^27^. Fetal fraction was estimated by taking average of the elastic net and the weighted rank selection criterion (WRSC) models from the SeqFF method using the autosomes.^29^ The dataset of fetal fraction used to plot the normal distribution in singleton pregnancies was the same presented in Brison et al.^19^, and correspond to a total of 21,982 pregnancies. GC-corrected normalized read counts were collected per 50 kb bin and aggregated per chromosome. Normalized sex chromosomes frequencies were calculated using X and Y chromosome read counts divided by the total number of read counts. No sample selection was performed based on fetal fraction. We applied two strategies—one-step multinomial logistic regression and two-step binomial logistic regression to predict twin sex. By using two-step binomial logistic regression, we reshape the twin sex discrimination in a multinomial logistic regression. The joint model fitting of multinomial logistic regression aggregates all cases that tend to perform less robust in stratified model fitting of two-step binomial logistic regression. Two-step multinomial logistic regressions were applied for twin sex discrimination based on the normalized frequencies of X and Y chromosomes, and fetal fraction estimation. A second-step regression was used after in the first-step a sample was classified as being non-female–female. The second run is applied in order to predict whether the twin sex is female–male or male–male. The regression analyses were done using R generalized linear model. By incorporating fetal fraction estimation into the regression model, the model performance improved. In particular, when discriminating female–male and male–male sex, the prediction was more associated with fetal fraction and normalized Y frequency changes. To test the predictive performance of this strategy, we further used a leave-one-out cross-validation strategy. We omitted one sample at each step and used the remaining samples to train the regression model. The trained model was then used to predict the sex of the sample that was left out. The leave-one-out cross-validation was performed using R pipelearner package, and plots were generated using ggplot2 and rgl packages. The sensitivity and specificity were calculated using the prediction results.

### Reporting summary

Further information on research design is available in the Nature Research Reporting Summary linked to this article.

## Supplementary information

Reporting Summary

Supplemental Legend File

Supplementary Movie 1

Supplementary Data 1

Supplementary Data 2

## Data Availability

The raw read counts obtained from massively parallel sequencing are available in Supplementary Data 2. The sequencing data is available to academic users upon request to the Data Access Committee of KU Leuven via the corresponding author (J.R.V.).

## Supplementary information

**Supplementary Information** accompanies the paper on the *npj Genomic Medicine* website (10.1038/s41525-019-0089-4).
