# Supplementary material for: Fetal sex determination in twin pregnancies using non-invasive prenatal testing
Source: NPJ Genom Med. 2019 Jul 4;4:15. doi: 10.1038/s41525-019-0089-4 (PMC6609680; doi:10.1038/s41525-019-0089-4)
Supplement: Supplementary file 2 — Supplemental Legend File [file 41525_2019_89_MOESM2_ESM.docx]

**Supplementary Movie 1. 3D rotation animation.** Correlation of the normalized frequencies of X and Y chromosomes, and fetal fraction (FF) incorporated into the multinomial logistic regression model.

**Supplementary Data 1.** Multinomial logistic regression data for fetal sex determination in twin pregnancies

**Supplementary Data 2.** Raw read counts obtained from NIPT sequencing data
