# Supplementary figures and images for: Fetal sex determination in twin pregnancies using non-invasive prenatal testing
Source: NPJ Genom Med. 2019 Jul 4;4:15. doi: 10.1038/s41525-019-0089-4 (PMC6609680; doi:10.1038/s41525-019-0089-4)

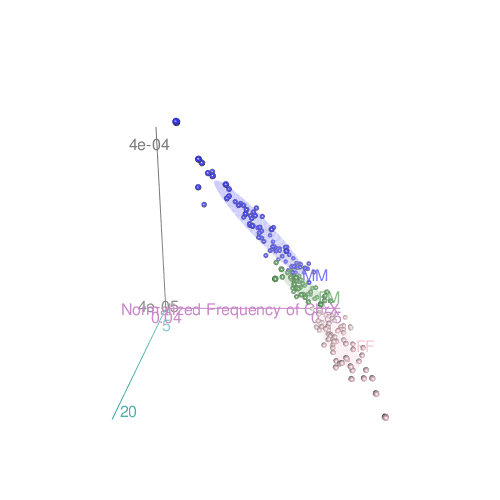

Supplement: Supplementary file 3 — Supplementary Movie 1 [file 41525_2019_89_MOESM3_ESM.gif]
